# Overview of the effect of rumen-protected limiting amino acids (methionine and lysine) and choline on the immunity, antioxidative, and inflammatory status of periparturient ruminants

**DOI:** 10.3389/fimmu.2022.1042895

**Published:** 2023-01-12

**Authors:** Muhammad Zahoor Khan, Shuai Liu, Yulin Ma, Mei Ma, Qudrat Ullah, Ibrar Muhammad Khan, Jingjun Wang, Jianxin Xiao, Tianyu Chen, Adnan Khan, Zhijun Cao

**Affiliations:** ^1^ State Key Laboratory of Animal Nutrition, Beijing Engineering Technology Research Center of Raw Milk Quality and Safety Control, College of Animal Science and Technology, China Agricultural University, Beijing, China; ^2^ Faculty of Veterinary and Animal Sciences, the University of Agriculture, Dera Ismail Khan, Pakistan; ^3^ Anhui Province Key Laboratory of Embryo Development and Reproduction Regulation, Anhui Province Key Laboratory of Environmental Hormone and Reproduction, School of Biological and Food Engineering, Fuyang Normal University, Fuyang, China; ^4^ Genome Analysis Laboratory of the Ministry of Agriculture, Agricultural Genomics Institute at Shenzhen, Chinese Academy of Agricultural Sciences, Shenzhen, China

**Keywords:** oxidative stress, periparturient period, ruminants, antioxidants, immunity, limiting amino acids

## Abstract

Overproduction of reactive oxygen species (ROS) is a well-known phenomenon experienced by ruminants, especially during the transition from late gestation to successful lactation. This overproduction of ROS may lead to oxidative stress (OS), which compromises the immune and anti-inflammatory systems of animals, thus predisposing them to health issues. Besides, during the periparturient period, metabolic stress is developed due to a negative energy balance, which is followed by excessive fat mobilization and poor production performance. Excessive lipolysis causes immune suppression, abnormal regulation of inflammation, and enhanced oxidative stress. Indeed, OS plays a key role in regulating the metabolic activity of various organs and the productivity of farm animals. For example, rapid fetal growth and the production of large amounts of colostrum and milk, as well as an increase in both maternal and fetal metabolism, result in increased ROS production and an increased need for micronutrients, including antioxidants, during the last trimester of pregnancy and at the start of lactation. Oxidative stress is generally neutralized by the natural antioxidant system in the body. However, in some special phases, such as the periparturient period, the animal’s natural antioxidant system is unable to cope with the situation. The effect of rumen-protected limiting amino acids and choline on the regulation of immunity, antioxidative, and anti-inflammatory status and milk production performance, has been widely studied in ruminants. Thus, in the current review, we gathered and interpreted the data on this topic, especially during the perinatal and lactational stages.

## 1 Introduction

Under normal physiological conditions, the antioxidant system’s capacity to neutralize and eliminate reactive oxygen species (ROS) produced during metabolic activities is usually sufficient. Metabolic alterations during pregnancy and calving have been shown to increase ROS generation above the level that the antioxidant system can cope with (1, 2). When there is an imbalance between the generation of ROS and the availability of antioxidant molecules, oxidative stress arises, exposing cattle to a variety of illnesses (3, 4). An excessive generation of ROS results in lipid peroxidation, oxidative stress, tissue damage, and changes in the quantity of reduced glutathione (GSH), a key component of glutathione metabolism (3, 5). When the pro/antioxidant balance is disrupted, damage to the structure and function of cellular macromolecules (lipids, proteins, and nucleic acids) occurs, resulting in oxidative stress. A preponderance of oxidation over reduction processes leads to metabolic disorders and diseases in dairy cows (6). Maintaining redox homeostasis in dairy cows during the periparturient and peak lactation stages is therefore critical (4, 7, 8). Parturition-related oxidative stress may contribute to immunological and inflammatory abnormalities, which increase the risk of metabolic and infectious disorders (2, 9).

**Figure 1:**
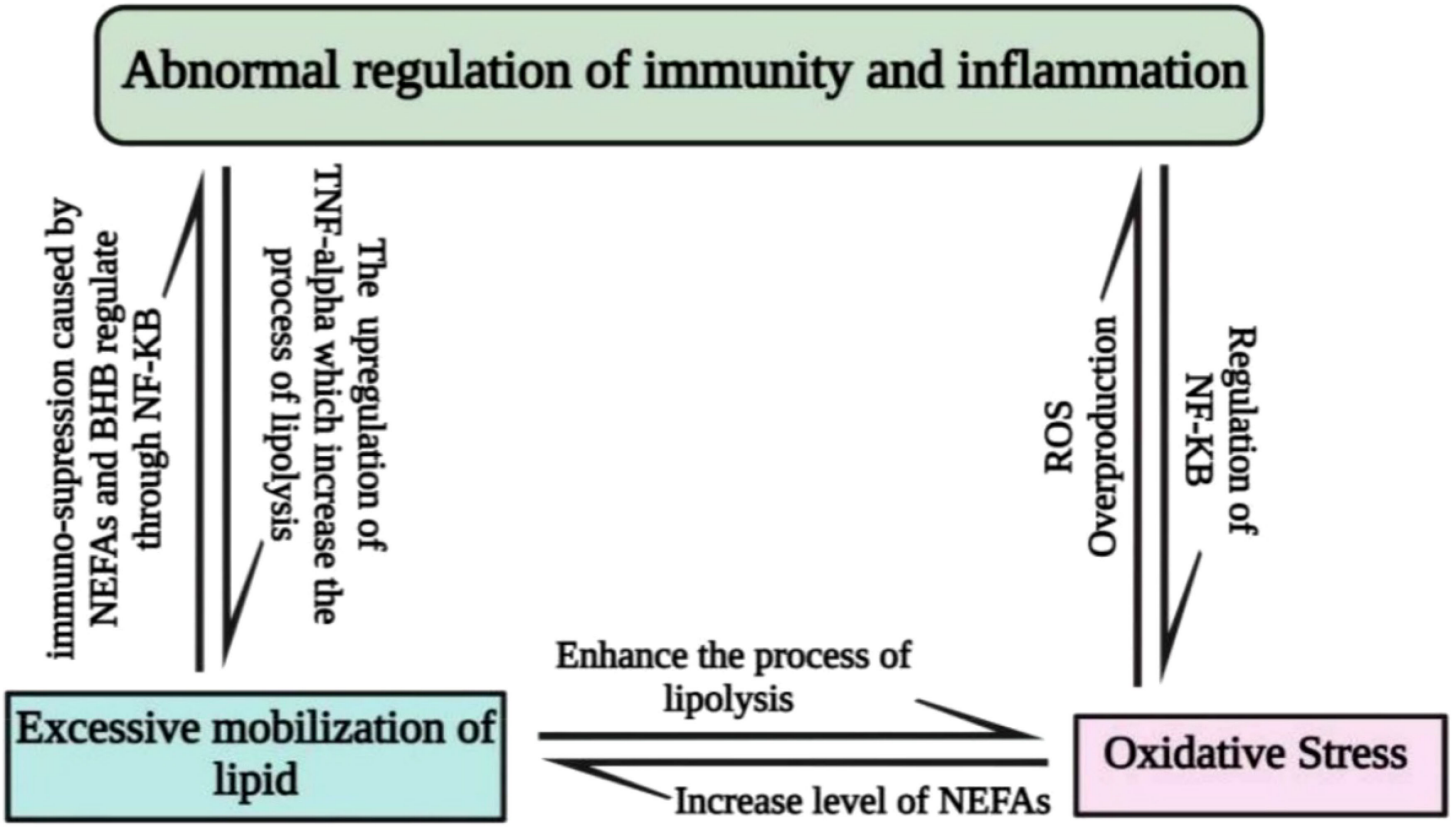
Interlink between metabolic stress, oxidative stress, immunity, and inflammation; nuclear factor kappa-B (NF-kB) signaling is used by oxidative stress to promote inflammation and immune system malfunction. Improper inflammatory regulation promotes the synthesis of tumor necrosis factor-alpha in non-phagocytic cells, leading to an excess of OS and excessive lipolysis. Oxidative stress increases the level of NEFAs, which is correlated with excessive lipid mobilization. The excessive lipid mobilization may elevate the level of NEFAs and BHB, which are the key factors in the abnormal regulation of the immune and inflammatory response.

Metabolic stress during the periparturient period is another key factor that exposes animals to immune depression, abnormal regulation of the inflammatory response, and oxidative stress. During the periparturient period, metabolic stress causes excessive mobilization of lipids followed by oxidative stress (10). The oxidative stress compromises the immunity and inflammatory status in dairy cattle, as shown in **Figure 1**.

Another critical factor is the abnormal regulation of immunity and inflammation caused by metabolic and oxidative stress (11–14), which predisposes dairy animals to various infections. In addition, the productive efficiency of animals is compromised by oxidative and metabolic stress, resulting in negative energy balance, immune suppression, and low productive efficiency (15–17). Several nutritional strategies have been adopted to overcome these issues during the periparturient period in ruminants.

The role of amino acids in the regulation of intestinal immunity and inflammation has been discussed previously (18). In brief, He et al. (18) reported that amino acid supplementation regulates the expression of anti-inflammatory cytokines and suppresses the apoptosis of enterocytes, as well as the expression of pro-inflammatory cytokines in intestinal inflammation. Furthermore, it was noted that several anti-inflammatory-related pathways, such as nuclear factor-kappa-B (NF-ϰB), mitogen-activated protein kinase (MAPK), inducible nitric oxide synthase (iNOS), mechanistic target of rapamycin (mTOR), nuclear erythroid-related factor 2 (Nrf2), and angiotensin-converting enzyme 2 (ACE2), are regulated by amino acid supplementation (18, 19).

Rumen-protected amino acid (methionine and lysine) and choline supplementation has been widely studied for its role in the maintenance of the antioxidant status, immunity status, and anti-inflammatory ability of dairy cattle (11, 20–25). Amino acids such as lysine, choline, and methionine are considered important sources of antioxidants in the diets of ruminants because of their positive role in relieving oxidative stress, which is associated with better health and productive efficiency (22, 23, 26). Thus, in the current review, we summarize the effect of rumen-protected first-limiting amino acids (methionine and lysine) and choline on the regulation of immunity, antioxidative, and anti-inflammatory status, especially during the periparturient period in dairy cattle in which they experience severe oxidative stress followed by depressed immunity and anti-inflammatory status.

## 2 Effect of rumen-protected first-limiting amino acids (methionine and lysine) and choline on the antioxidant, immunity, anti-inflammatory, and health status of ruminants

### 2.1 Effect of rumen-protected first-limiting amino acids (methionine and lysine) and choline supplementation on the antioxidant status of ruminants

#### 2.1.1 Effect of methionine and choline supplementation on the antioxidant status of ruminants

The availability of key methyl donors, such as methionine and choline, is limited due to extensive microbial degradation in the peripartal dairy cow (27). If, for any reason, an imbalance of negative methyl donor occurs it will compromise the synthesis of critical compounds, such as carnitine and phosphatidylcholine (PC), which are required for methyl donors in cells and tissues (28). The biosynthesis of PC from endogenous supplementation of methionine and choline could prevent the incoming fatty acids produced from the lipolysis of adipose tissue. It has been documented that the disproportionate infiltration of hepatic fatty acid can negatively influence the normal function of the liver (29). Moreover, the excessive fat infiltration in the liver may suppress intracellular antioxidants, such as glutathione (GSH) and alternatively oxidative stress, which may lead to extensive tissue damage (11, 30, 31). The methyl donors are considered the most important extracellular source of the antioxidants glutathione (GSH) and taurine (32). During the periparturient period, the increased oxidative stress enhances inflammation, resulting in suppressed leukocyte responses. To overcome these challenges and for cells to synthesize sulfur-containing antioxidants, the supplementation of methionine and choline could be beneficial.

Rumen-protected limiting amino acids, such as methionine, are necessary for the biosynthesis of S-adenosylmethionine (SAM) (33). SAM is required for many biological processes, such as transsulfuration, polyamine biosynthesis, and DNA methylation (34). Thus, the need for methyl donors, including choline and methionine, is increased at the onset of the lactation period (35, 36). It has been well-documented that DNA methylation regions are associated with the regulation of gene expression. These epigenetic changes are partly driven by betaine and methionine through SAM. A study documented that rumen-protected choline enhances the expression of genes associated with the synthesis of betaine, phosphatidylcholine, acetylcholine, muscarinic, and nicotinic acetylcholine receptors (37). Interestingly, betaine has been found to work as a methyl donor and help in the regeneration of methionine and SAM (38). In addition, phosphatidylcholine has been found to be involved in the export of low-density protein, which is followed by a decrease in hepatic fat accumulation (39). Furthermore, a study found that methionine supplementation is necessary for the regulation of the nuclear receptor peroxisome proliferator-activated receptor alpha (PPARα), which plays a key role in reducing the inflammatory response, improving fatty acid oxidation, and preventing liver triacylglycerol (TAG) accumulation (40).

According to the available literature, rumen-protected choline and methionine are considered as valuable antioxidants in ruminant nutrition (41). Recent evidence suggests that methionine plays a role in glutathione formation (42), which could improve the antioxidant status of both animals and their products. Consistently, a study reported that methionine alone or in combination with choline alleviates oxidative stress by enhancing glutathione transferase (GST) activity in the plasma of periparturient ewes (43). Furthermore, another study documented that methionine in combination with choline enhances glutathione and amino acid levels in dairy cows during the perinatal period (44).

#### 2.1.2 Effect of methionine and lysine supplementation on the antioxidant status of ruminants

Methionine is attacked by many of the ROS generated in biological systems and is considered one of the more readily oxidized residues in proteins (45–47). Methionine has a scavenging ability, and various ROS combine with methionine to form methionine sulfoxide. The cells contain methionine sulfoxide reductases, which catalyze the thioredoxin-dependent reduction of methionine sulfoxide back to methionine (47). Methionine acts as a catalytic antioxidant and protects protein and macromolecules from the action of ROS in which they are present.

The antioxidant role of lysine and methionine has been widely studied in ruminants (48–51). Furthermore, it has been highlighted that amino acids enhance metabolism, antioxidant status, and immunity to improve production and disease resistance. Consistent with this, it has been well established that amino acids play a key role in cellular oxidative balance (52) because they participate in taurine and glutathione (GSH) biosynthesis (53–55). GSH using glutathione transferase (GST) and hydrogen peroxide neutralization *via* glutathione peroxidase (GSH-Px) causes cellular detoxification. Glutathione transferases (GSTs, EC 2.5.1.18) are some of the most important antioxidant enzymes for regulating the cell’s redox state (56–58). Similarly, a reduced level of glutathione (a potent intracellular antioxidant) was reported in liver tissue in response to methionine treatment (59).

It has been reported that methionine supplementation enhances very low-density lipoprotein (VLDL) to promote vitamin E in circulation (60). Consequently, the detrimental effect of lipid peroxidation by-products, such as malondialdehyde (MDA), can be suppressed with supplementation with rumen-protected amino acids (60). Furthermore, antioxidant systems, which can be split into enzymatic and non-enzymatic (e.g., metabolites), control ROS (56, 61). The effect of methionine and lysine supplementation on oxidative stress has been studied in ewes (21). It was further noted that methionine supplementation reduces the expression of MDA and improves the level of superoxide dismutase (SOD), CAT, GPH, and GST in the plasma of ewes (21).

A few studies, through *in vitro* experiments, reported that methionine supplementation decreases the process of apoptosis and necrosis and inhibits lipid peroxidation in the bovine mammary glands (62, 63). Moreover, they documented that methionine supplementation enhances the level of antioxidant-associated genes (superoxide dismutase and glutathione peroxidase) and the cytoprotective effect against hyperthermia (62, 63).

### 2.2 Effect of methionine and lysine supplementation on inflammation and immunity in ruminants

Parturition has been characterized as the stage of inflammatory change during which the level of haptoglobin is elevated, thus decreasing the concentration of albumin (64). Moreover, albumin levels have been reported to decrease around parturition and are also considered as a key biomarker for inflammation in dairy cattle. The higher the level of albumin, especially during the periparturient period, the healthier the animal (65). A higher concentration of albumin has been reported in response to methionine supplementation in dairy cattle (64). Moreover, by binding to non-esterified fatty acids and bilirubin, albumin takes on a detoxifying function and alleviates inflammation (66), which is the critical factor that exposes periparturient dairy cattle to infections. Haptoglobin is another key acute-phase protein, which usually increases around parturition because of inflammatory events and parenchymal cell stimulation by fatty acid infiltration of the liver. Furthermore, it has been demonstrated that methionine supplementation significantly downregulates the level of haptoglobin, which is associated with improved antioxidant and anti-inflammatory levels during parturition in dairy cattle (64). Furthermore, Zhou et al. (64) found that dairy cattle supplemented with methionine were less susceptible to infections during the periparturient period because of suppressed inflammatory changes and improved antioxidant status.

Previous studies have proved experimentally that methionine supplementation enhances the anti-inflammation and anti-oxidative status in periparturient dairy cattle (59, 67, 68) and neonatal calves (69). In a recent study, Hu et al. reported that methionine and arginine significantly regulates milk protein synthesis, thereby alleviating a potential inflammatory and pro-oxidant state in transitioning dairy cattle (70). In addition, a few studies have reported that the supplementation of methionine with arginine plays an important role in anti-inflammation and improves the antioxidant status of transition dairy cattle (68, 71). Moreover, methionine and arginine supplementation has been associated with the regulation of immunity and relief of oxidative stress caused by bacterial lipopolysaccharide (LPS) in bovine mammary cells (71).

It has been reported that rumen-protected lysine and methionine feeding before parturition to transition dairy cows may affect the immunity of calves (69). In an experimental trial, it has been reported that the offspring (calves) of rumen-protected methionine and lysine-fed cows show higher passive immunity, including a higher concentration of immunoglobulin G, higher serum total protein, and a higher growth rate than an unsupplemented group of dairy cows (69). Lee et al. (72) reported that supplementation with lysine and methionine positively reduces milk somatic cell count (SCC) and improves the immunity and health status of dairy cattle. Consistent with this, a study proved experimentally that methionine supplementation tends to decrease metabolic stress and milk SCC and improve udder health in goats (73).

### 2.3 Effect of methionine and choline supplementation on the inflammation and immunity of ruminants

It has been well established through experimental trials that treatment with a combination of choline and methionine can regulate the antioxidative state, thereby increasing the anti-inflammatory and cytoprotective effect against oxidative stress in neonatal Holstein calves (74). Consistent with this, a study reported that supplementation with choline and methionine improves the immunometabolic state, blood polymorphonuclear leukocyte phagocytosis capacity, and the anti-inflammatory effect upon pathogen challenge, and enhances the antioxidative capacity of peripartal cows (75). During the periparturient period, the liver functionality index (LFI) is the key indicator that is used to assess immune and inflammatory status, as well as metabolic profiles in dairy cattle (44, 59, 76). In addition, a study reported that a low LFI and low amino acids in circulating plasma indicates a difficult transition from gestation to lactation (30). The supplementation of a combination of choline and methionine enhances the level of the LFI, resulting in an improved immunity and anti-inflammatory status in dairy cattle (44). Moreover, ketosis was also found to be positively associated with a low LFI, while combined supplementation of choline and methionine significantly reduces the chances of ketosis by alleviating oxidative stress in dairy cattle (77).

Choline supplementation in the transition diet may be partially useful for reducing the deleterious effects of an inflammatory-like condition on the hepatic function of transition cows. The non-esterified fatty acids (NEFA) and β-hydroxybutyric acid (BHB) may lead to oxidative stress followed by an enhancement of the lipolysis process (78). Additionally, rumen-protected choline supplementation has been found to reduce the concentration of liver triacylglycerol and metabolic stress, resulting in an improved immune and antioxidative state (79, 80). Consistent with this, choline supplementation also lowers BHBH levels and the body condition score, which is the best indicator of health in dairy cattle during the periparturient period (81, 82). Furthermore, it has been reported that excessive production of NEFA and BHB may compromise immunity, resulting in abnormal regulation of immune and inflammation responses (83). The high levels of NEFA and BHB followed by oxidative stress and dysfunctional immunity have been reported as major contributory factors of mastitis in dairy cattle (10). Several studies have demonstrated that choline supplementation significantly reduces the level of NEFA and BHB, resulting in enhanced immunity and maintenance of the anti-inflammatory state in dairy cows (84–87).

During early lactation, catabolic changes are initiated and lead to the utilization of body energy reserves (non-esterified fatty acids and amino acids). The excessive utilization of NEFA is incorporated into VLDL by the liver. A high level of VLDL is associated with oxidative stress and fatty liver syndrome (88). Furthermore, supplementation of rumen-protected choline enhances the function of the liver by improving VLDL exportation from the liver and relieving oxidative stress (88). By increasing the expression of fatty acid transport protein 5 and carnitine transporter *SLC22A5* in the liver and endorsing apo B-containing lipoprotein assembly, rumen-protected choline supplementation reduces the harmful effects of hepatic lipidosis in periparturient dairy cattle (89). Consistent with this, a study reported that choline significantly decreases the level of postpartal liver TAG and enhances the biosynthesis of VLDL through the regulation of PPARα targets apolipoprotein B (*APOB*) and microsomal triglyceride transfer protein (*MTTP*) (89).

It has been well demonstrated that heat stress compromises immunity and regulates the inflammatory system in an abnormal way, affecting the production performance of dairy cattle (90, 91). Nutritional intervention with methionine and choline has been reported to enhance the immune response to heat stress and play a key role in improving the health of animals (92–94). Furthermore, choline supplementation improves lipid and energy metabolism and alleviates the inflammatory response (95). Rumen-protected choline feeding during the periparturient period is the key focus because of its key role in preventing liver lipid accumulation through VLDL export. Moreover, it has been reported that choline plays an important role in the regulation of immune function and also mitigates inflammatory changes in transition dairy cows (93), enhances immune function in calves (94), and adjusts the responses of immune cells to LPS *ex vivo* (37). Consequently, it has been documented that choline deficiency may lead to intestinal morphology and lipid metabolism impairment in rats (96). The antioxidant, anti-inflammatory and immune regulatory role of limiting amino acids (methionine and lysine) and choline has been summarized in **Table 1**. Based on the available data, we concluded that choline supplementation maintains the inflammatory response in immune cells and relieves metabolic stress during the periparturient period in dairy cattle.

**Table 1:** Summary of studies in dairy ruminants investigating the influence of rumen-protected limiting amino acids (methionine and lysine) and choline on immune function and the oxidative and anti-inflammatory status of ruminants.

Amino acid supplementation	Main outcomes	Species/tissues/cells	Author
Rumen-protected methionine supplementation	Alleviates inflammation and oxidative stress, resulting in improved liver function Enhances oxidative burst and neutrophil phagocytosis	Plasma (cattle)	Batistel et al. (68)
Methionine and choline supplementation	Methionine is utilized by cells for the synthesis GSH and taurine synthesis Increases metabolism and decreases oxidative stress, inflammation, and enhanced immunity Improves liver function Improves oxidative burst and neutrophil phagocytosis	Liver and plasma (Dairy cattle)	Zhou et al. (59); Zhou et al. (64)
Methionine supplementation	Enhances antioxidant status and improves the immune and anti-inflammatory response in periparturient dairy cattle	Dairy cattle	Zhou et al. (44)
Methionine supplementation	Decreases inflammation and enhances immunity during a transition period and reduces the chances of infection	Dairy calves	Jacometo et al. (97)
Methionine and choline supplementation	Decreases inflammation and enhances immunity during a transition period, and reduces the chances of infection	Dairy calves	Abdelmegeid et al. (74)
Methionine or lysine supplementation	Enhances innate immunity Decreases inflammation and relieved oxidative stress	Sheep	Tsiplakou et al. (43); Tsiplakou et al. (98)
Zinc and methionine supplementation	Decreases inflammation, lowers milk SCC, and prevents mastitis by enhancing immunity and alleviating inflammation	Goats	Salama et al. (73)
Choline and methionine supplementation	Enhances CD4^+^/CD8^+^ T lymphocyte ratio and improves immunity and anti-oxidative status Regulates hepatic lipid metabolism and relief of metabolic stress Improves immunity	Dairy cattle	Sun et al. (60)
Hydroxyselenomethionine supplementation	Increases antioxidant response followed by the relief of oxidative status and improved anti-inflammatory response in dairy cattle during the periparturient period	Dairy cattle	Li et al. (99)
*N*-acetyl-l-methionine supplementation (NALM)	Relieves oxidative stress in lactating dairy cows Increases concentrations of total protein and globulin in plasma and significantly reduces plasma malonaldehyde concentration	Dairy cattle	Liang et al. (100)
NALM supplementation	Improves metabolism and enhances milk production	Dairy cattle	Fagundes et al. (101)
Methionine supplementation	Upregulates the expression of genes involved in the metabolism of antioxidants Increases the expression of NFE2L2 (a major antioxidant transcription factor) and improves the immune and anti-inflammatory response	Dairy cattle	Han et al. (48)
Methionine supplementation	Enhances mRNA expression of genes related to antioxidative status and GSH metabolism	Dairy cattle	Liang et al. (100)
Methionine and choline supplementation	Enhances the expression of genes associated with the immune and anti-inflammatory response and reduces oxidative stress	Dairy cattle	Lopreiato et al. (102)
Methionine supplementation	Improves whole-blood neutrophil phagocytosis and decreases oxidative stress, resulting in enhanced immunity	Dairy cattle	Osorio et al. (103)
Methionine supplementation	Enhances 1-carbon metabolism and increases the antioxidative status. In addition, methionine increases liver GSH and decreases concentrations of plasma biomarkers of inflammation.	Dairy cattle	Osorio et al. (42); Osorio et al. (67)
Methionine and choline supplementation	Blocks the hyper response of IL-1β during LPS challenge, resulting in improved antioxidant ability	Dairy cattle	Vailati-Riboni et al. (104)
Methionine supplementation	Increases the expression of genes associated with immunity and antioxidant response	Dairy cattle	Zhou et al. (105)
Methionine supplementation	The expression of inflammation- and oxidative stress-associated genes is significantly reduced by methionine supplementation	Dairy cattle	Zhou et al. (75)
Methionine supplementation	Enhances the metabolism of carnitine and β-oxidation of fatty acids and improves cholesterol and lipoprotein metabolism Improves 1-carbon metabolism of cystathionine Beta-synthase activity of cystathionine followed by enhancement of antioxidant synthesis	Dairy cattle	Zhou et al. (105); Alharthi et al. (106)
Choline supplementation	Alleviates fatty liver and relieves metabolic stress	Dairy cattle	Guretzky et al. (107)
Rumen-protected choline and methionine supplementation	Decreases oxidative stress, followed by enhancement of antioxidant capacity and improved immunity	Sheep	Tsiplakou et al. (43)
Choline supplementation	Decreases liver triacylglycerol concentration of plasma and improves immunity Reduces the incidence of subclinical hypocalcemia	Dairy cattle	Bollatti et al. (108)

### 2.4 Rumen-protected limiting amino acids (methionine and lysine) and choline-regulated genes are associated with the immunity, antioxidant, and anti-inflammatory status of ruminants

The molecular response associated with health regulation in response to rumen-protected limiting amino acids in dairy ruminants has been well documented (21). It has been reported that heat stress upregulates miR-34a, miR-92a, miR-99, and miR-184. In addition, the upregulation of microRNA in the mammary gland by heat stress is related to cell growth arrest and apoptosis and inhibition of fat synthesis (109). Consistent with this, it has been documented that miR-34a overexpression induces apoptosis in Hep2 cells (110) and mammary cells (111) and inhibits cell proliferation (110). Salama et al. proved through experimental trials that methionine and arginine supplementation downregulates the expression of miR-34a, miR-92a, miR-99, and miR-184 in the mammary gland. Furthermore, they documented that methionine and arginine treatment regulates most of the genes that are associated with insulin signaling (*AKT2* and *IRS1*), transcription and translation (*MAPK1*, *MTOR*, *SREBF1*, *RPS6KB1*, and *JAK2*), amino acid transport (*SLC1A5* and *SLC7A1*), and cell proliferation (*MKI67*) in the mammary glands of dairy cattle (109). Heat stress also upregulates genes associated with apoptosis (*BAX)*, translation inhibition (*EIF4EBP1*), and lipogenesis (*PPARG*, *FASN*, and *ACACA*) and decreases the expression of the anti-apoptotic gene *BCL2L1* in the mammary glands of dairy cattle (109). The above effects caused by heat were reversed with methionine and arginine supplementation in dairy cattle (109).

**Figure 2:**
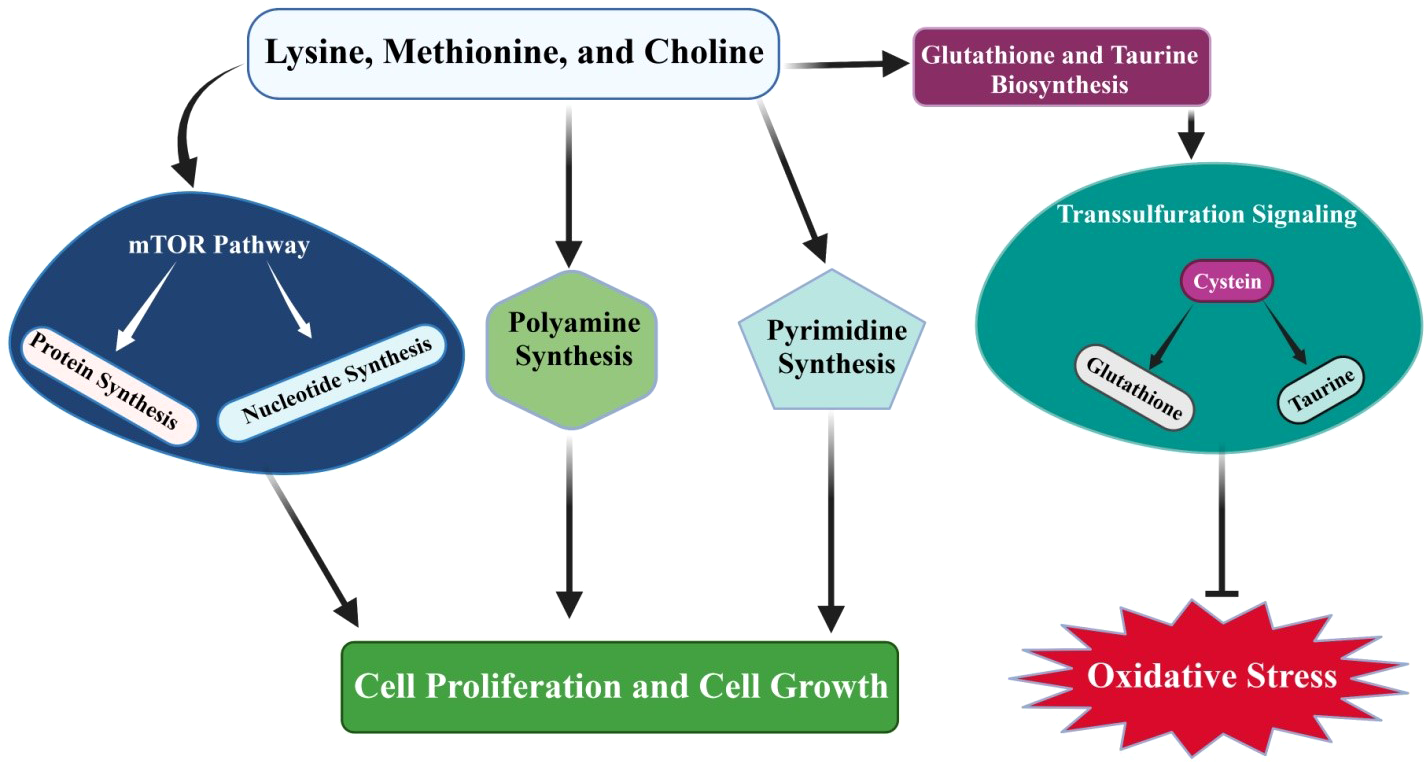
Rumen-protected limiting amino acids (lysine and methionine) and choline supplementation regulate mTOR signaling, which controls protein and nucleotide synthesis. In addition, amino acids mediate transsulfuration pathways that further facilitate taurine and glutathione biosynthesis. Taurine and glutathione biosynthesis plays a key role in alleviating oxidative stress in dairy cattle. Additionally, amino acid supplementation regulates mTOR signaling and pyrimidine and polyamine synthesis, and thus plays an essential role in controlling cell proliferation and growth processes.

71, reported that bacterial LPS significantly downregulates the expression of *SLC36A1* and *SLC7A1* and genes associated with antioxidant response (*NFE2L2*, *NQO1*, *GPX1*, *ATG7*, and *GPX3*), and upregulates *SOD2* and *NOS2* (71). Furthermore, they noticed that arginine and methionine supplementation enhances antioxidative gene expression and increases the signaling level of *NFE2L2* in mammary gland cells. Ma et al. (112) found that *NFE2L2* signaling plays a critical role in the cellular antioxidant defense system. It might be possible that arginine and methionine treatment significantly enhances the antioxidative response in mammary gland cells *via* the *NFE2L2* pathway (112). Consistent with this, it has been found that methionine-treated mammary gland cells show increased expression of *CSN1S1*, *CSN1S2*, *CSN2*, *CSN3*, *LALBA*, *JAK2*, *STAT5*, and *MTOR*, which are positively linked to milk protein synthesis (113, 114). Methionine supplementation significantly regulates the transsulfuration pathways that play a key role in taurine and glutathione biosynthesis. Taurine and glutathione biosynthesis alleviates the oxidative stress that is caused by negative energy balance in neonatal Holstein calves (115), as demonstrated in **Figure 2**.

Garcia et al. experimentally proved that choline supplementation regulates the genes that are associated with muscarinic and nicotinic acetylcholine (37). The muscarinic and nicotinic acetylcholine receptors have been detected in innate and adaptive immune cells (116). Garcia et al. identified several genes involved in choline metabolism (*SLC5A7, CHDH, CHKA, ACHE, CHRM5*, and *CHRNA7*) and inflammatory responses (*TLR4, NFKB1, TNFA, ELANE, H2A, CASP3*, and *CASP7*) in the neutrophils and monocytes of lactating dairy cattle, which are regulated in response to rumen-protected choline supplementation (37). Inflammatory genes (*TLR4*, *NFKB1*, and *TNFA*) are significantly upregulated in LPS-challenged immune cells; however, the opposite trend is observed for these genes in choline-treated immune cells (37).

Methionine supplementation elevates the expression of genes associated with inflammation (*IL1B*, *TLR2*, *NF-κB*, and *STAT3*) and oxidative stress (glutathione synthase, *GPX1*, *CBS*, and *SOD2*) in polymorphonuclear leukocytes (PMNLs) and enhances taurine in the plasma of dairy cattle (75). These findings suggest that methionine supplementation improves the anti-inflammatory and antioxidative status of periparturient dairy cattle. Furthermore, PMNLs treated with LPS reveal the hyper response of IL-1β, resulting in oxidative stress (104). Rumen-protected methionine supplementation suppresses the hyper response of IL-1β, resulting in a decreased inflammatory response and oxidative stress (104). Consistent with this, it has been documented that the combination of methionine and choline significantly upregulates the expression of genes involved in pathogen recognition mechanisms (*TLR2* and L-selectin [*SELL*]) and lowers the expression of genes associated with inflammation (cysteine sulfinic acid decarboxylase [*CSAD*], cystathionine gammalyase [*CTH*], myeloperoxidase [*MPO*], glutathione reductase [*GSR*], *GSS*, *IL6*, *IL10*, and *IL1B*), resulting in an improved anti-inflammatory and antioxidative status in dairy cattle (102). Additionally, methionine downregulates the expression of several genes potentially associated with oxidative stress (*SOD1*, *GSS*, and *GCLC*) (42). Furthermore, Osorio et al. (42) reported higher expression of haptoglobin (*HP*), *S*-adenosylhomocysteine hydrolase (*SAHH*), adenosyltransferase 1A (*MAT1A*) cytosine-5-methyltransferase 3 alpha (*DNMT3A*), and *DNMT1* in response to methionine supplementation. Furthermore, they reported lowered oxidative stress and a mild inflammatory status in dairy cattle (42). Consistent with this, methionine and lysine combination treatment significantly reduces the level of TLR4, pro-inflammatory cytokines (TNF-α and IL-1β), chemokine (CXCL-16), and BHBA content in ewes (117). The summary of the genetic response to rumen-protected limiting amino acids (lysine and methionine) and choline is shown in **Table 2**.

**Table 2:** Summary of studies that investigated the rumen-protected limiting amino acid (methionine and lysine)- and choline-regulated genes associated with the immunity, antioxidative, and anti-inflammatory status of dairy ruminants.

Treatment	Genetic effect	Biological function	Species/tissues	Author
Methionine supplementation	Increases expression of *NFE2L2*, *NQO1*, *GPX1*, *GPX3*, *SLC36A1*, *SLC7A1*, *SOD2*, and *NOS2* and decreases expression of *RELA*, *IL1B*, *NF-kb*, and *CXCL2*	Induces anti-inflammation and anti-oxidative responses against LPS	BMECs in dairy cattle	Dai et al. (71)
Methionine supplementation	Upregulates the expression of AKT1 and mTORC1 signaling, *PPARG*, *FASN*, *ACACA*, *BCL2L1*, *MAPK1*, *MTOR*, *SREBF1*, *RPS6KB1*, *JAK2*, and *MKI67* and downregulates the expression of *HSPA1A*, *BAX*, and *EIF4EBP1*	Alleviates oxidative status and regulates immunity and anti-inflammatory status	BMECs in dairy cattle	Salama et al., (109)
Methionine and lysine supplementation	Upregulates JunD and downregulates the expression of genes associated with inflammation (*IL-1α*, *MyD88*, *STAT-3*, and *IL-10*)	Enhances innate immunity, resulting in a reduction of inflammatory changes	Ewes	Tsiplakou et al. (98)
Methionine and lysine supplementation	Lowers the expression of pro-inflammatory cytokines (IL-1β and TNF-α), the chemokine CXCL-16, and pathogen recognition receptor-4 (TLR-4) and suppresses β-hydroxybutyric acid (BHBA) content	Reduces inflammatory changes and enhances the immune response, resulting in the alleviation of mastitis	Ewes	Tsiplakou et al. (117)
Methionine and choline supplementation	Unregulates *GCLC, GPX1*, and adenosylhomocysteinase (*SAHH)* and lowers the expression of *CXCR1*, *IL10*, *IL6*, *IRAK1*, *NFKB1*, *NR3C1*, *SELL*, *TLR4*, and *TNFA* in polymorphonuclear leukocytes	Increases homocysteine synthesis, resulting in enhanced antioxidative status, and reduces inflammatory changes and cytoprotection against oxidative stress	Neonatal Holstein calves	Abdelmegeid et al. (74)
Choline supplementation	Decreases the expression of pro-inflammatory cytokines *IL1B*, *CXCL8*, and *TNF* caused by LPS in peripheral blood leucocytes Enhances the level of blood neutrophils undergoing phagocytosis and oxidative burst	Decreases inflammation and improves immunity	Holstein cows	Zenobi et al. (93)
Methionine supplementation	Reduces the level of oxidative stress associated with *MDA*, *CAT*, and *SOD* Enhances the level of antioxidant genes (*GST* and *GPH*)	Plays a critical role in the regulation of antioxidant activity against oxidative stress	Ewes	Mavrommatis et al. (21)
Methionine supplementation	Lowers the expression of genes related to inflammation (*IL1B*, *TLR2*, *NF-*κ*B*, and *STAT3)* Reduces the expression of genes associated with oxidative stress *(*glutathione synthase, *GPX1 CBS*, and *SOD1)*	Improves anti-inflammatory and antioxidative status	Dairy cattle	Zhou et al. (75)
Methionine and choline supplementation	Enhances the expression of *SELL* and *TLR2* Lowers the expression of *CSAD*, *CTH*, *MPO*, *GSR*, *GSS*, *IL6*, *IL10*, and *IL1B*	Improves the antioxidant ability and suppresses inflammatory changes	Dairy cattle	Lopreiato et al. (102)
Rumen-protected choline supplementation	Decreases the expression of genes associated with inflammation (*TLR4, NFKB1*, *TNFA*, *ELANE*, *H2A*, *CASP3*, and *CASP7*) in neutrophils and monocytes treated with LPS	Decreases inflammation Improves the health of periparturient dairy cattle and alleviates mastitis	Dairy cattle	Garcia et al. (37)
Methionine supplementation	Reduces the level of inflammation-associated genes (*SELL*, *CXCR2*, *NFKBIA*, *MYD88*, *TLR4*, *TLR2*, *GSS*, *GPX1*, *TNF*, and *IL1B*), and suppresses genes involved in the oxidative stress (*MPO* and *SOD1*) of polymorphonuclear leukocytes Enhances the level of antioxidant-linked genes (*NFE2L2* and *NOS2*)	Improves immunity and antioxidative ability and reduces inflammatory alterations	Holstein calves	Jacometo et al. (97)
Methionine supplementation	Enhances the level of methionine adenosyltransferase 1A (*MAT1A*), glutamate-cysteine ligase (*GCLC*), glutathione reductase (*GSR*), adenosylhomocysteinase (*AHCY*; also known as *SAHH*), and DNA (cytosine-5-)-methyltransferases (*DNMT1*, *DNMT3A*, and *DNMT3B*) Lowers the expression of cysteine sulfinic acid decarboxylase (*CSAD*)	Decreases oxidative stress, enhances immunity, and reduces metabolic stress, which is responsible for abnormal regulation of immunity and inflammation	Holstein calves	Jacometo et al. (115)
Methionine supplementation	Upregulates peroxisome proliferator- activated receptor alpha (PPARα)-associated genes (*ANGPTL4*, *FGF21*, and *PCK1)*	Upregulation of hepatic PPARA has been reported to be associated with lipid metabolism and immune function	dairy cattle	Osorio et al. (118)
Rumen-protected methionine	Regulates PPARα, fatty acid Desaturase 2 (*FADS2*), CBS, glutathione S-transferase omega 1 (*GSTO1*), *GPX1*, MAPK and MTOR activator *LAMTOR2*, mammary serum amyloid (*SAA*), peroxisome proliferator-activated receptor gamma (*PPARG*), and forkhead box O1 (*FOXO1*)	Enhances cell metabolism, reduces metabolic and oxidative stress, relieves inflammation and enhances immunity in Holstein cows	Holstein cows	Palombo et al. (119)
Methionine supplementation	Upregulates the expression of genes associated with amino acid transport (*SLC38A1*, *SLC38A2*, and *SLC7A1*) and valyl-tRNA (*VARS*), isoleucyl-tRNA synthetases (*IARS*), glucose transport solute carrier family 2 member 3 (*SLC2A1*), glucose transport solute carrier family 2 member 3 (*SLC2A3*), and casein α-s1 (*CSN1S1*) Elevates the expression of Janus kinase 2 (*JAK2*) and the phosphorylation status of AKT, protein phosphatase 1, and regulatory subunit 15A (*PPP1R15A*)	Enhances metabolism, increases the level of plasma amino acids, and decreases metabolic stress and inflammation Improves milk production	Holstein cows	Ma et al. (112)

## 3 Conclusions

Based on published data, we conclude that rumen-protected limiting amino acids (lysine and methionine) and choline supplementation alleviate oxidative stress, which is the primary cause of several diseases in ruminants. Moreover, oxidative stress, particularly during the periparturient period, compromises immunity, production performance, and metabolism in dairy ruminants. The supplementation of rumen-protected amino acids and choline, especially during the periparturient period, enhances antioxidative ability, resulting in the regulation of immunity and anti-inflammation status in dairy ruminants. Thus, supplementation with a sufficient quantity of rumen-protected amino acids (lysine and methionine) and choline is highly recommended for protecting animals from diseases and enhancing their productive abilities.

## Author contributions

MK, MM, and ZC designed the study and wrote the manuscript; ZC supervised the manuscript; MM, YM, JW, QU, JX, TC, SL, IMK and AK helped in collection of data resources and editing of final version of manuscript. All authors contributed to the article and approved the submitted version.

## References

[1] Sordillo LM. Factors affecting mammary gland immunity and mastitis susceptibility. Livest Prod Sci (2005) 98:89–99. doi: 10.1016/j.livprodsci.2005.10.017

[2] Mavangira V Sordillo LM. Role of lipid mediators in the regulation of oxidative stress and inflammatory respons-es in dairy cattle. Res Vet Sci (2018) 116:4–14. doi: 10.1016/j.rvsc.2017.08.002 28807478

[3] Mikulková K Kadek R Filípek J Illek J. Evaluation of oxidant/antioxidant status, metabolic profile and milk production in cows with metritis. Irish Vet J (2020) 73(2020):1–11. doi: 10.1186/s13620-020-00161-3 PMC7254763 32514335

[4] Castillo C Hernandez J Valverde I Pereira V Sotillo J Alonso ML. Plasma malonaldehyde (MDA) and total antioxidant status (TAS) during lactation in dairy cows. Res vet Sci (2006) 80(2):133–9. doi: 10.1016/j.rvsc.2005.06.003 16084546

[5] Wullepit N Raes K Beerda B Veerkamp RF Fremaut D De Smet S. Influence of management and genetic merit for milk yield on the oxidative status of plasma in heifers. Livest Sci (2009) 123(2-3):276–82. doi: 10.1016/j.livsci.2008.11.013

[6] Sharma N Singh N Singh O Pandey V Verma P. Oxidative stress and antioxidant status during transition period in dairy cows. Asian-Australas J Anim Sci (2011) 24:479–84. doi: 10.5713/ajas.2011.10220

[7] Kowalska J Jankowiak D. Changes of reduction-oxidation balance in pregnant ruminants. Postepy biochemii (2009) 55(3):323–8. 19928589

[8] Markiewicz H Gehrke M Malinowski E Kaczmarowski M. Evaluating the antioxidant potential in the blood of tran-sition cows. Med Weterynaryjna (2005) 61:1382–4.

[9] Mavangira VMJ Mangual JC Gandy L Sordillo M. 15-F_2t_-Isoprostane concentrations and oxidant status in lactating dairy cattle with acute coliform mastitis. J Vet Intern Med (2016) 30:339–47. doi: 10.1111/jvim.13793 PMC4913657 26566597

[10] Khan MZ Ma Y Xiao J Chen T Ma J Liu S. Role of selenium and vitamins e and B9 in the alleviation of bovine mastitis during the periparturient period. Antioxidants (2022) 11(4):657. doi: 10.3390/antiox11040657 35453342 PMC9032172

[11] Sordillo LM Aitken SL. Impact of oxidative stress on the health and immune function of dairy cattle. Vet Immunol Immunopathol (2009) 128:104–9. doi: 10.1016/j.vetimm.2008.10.305 19027173

[12] Agrawal A Khan MJ Graugnard DE Vailati-Riboni M Rodriguez-Zas SL Osorio JS. Prepartal energy intake alters blood polymorphonuclear leukocyte transcriptome during the peripartal period in Holstein cows. Bioinform Biol Insights (2017) 11:1177932217704667. doi: 10.1177/1177932217704667 28579762 PMC5414586

[13] Mann S Sipka AS Grenier JK. The degree of postpartum metabolic challenge in dairy cows is associated with peripheral blood mononuclear cell transcriptome changes of the innate immune system. Dev Comp Immunol (2019) 93:28–36. doi: 10.1016/j.dci.2018.11.021 30500340

[14] Mann S Sipka A Leal Yepes FA Nydam DV Overton TR Wakshlag JJ. Nutrient-sensing kinase signaling in bovine immune cells is altered during the postpartum nutrient deficit: a possible role in transition cow inflammatory response. J Dairy Sci (2018) 101:9360–70. doi: 10.3168/jds.2018-14549 30055922

[15] Yin FG Zhang ZZ Ju H Yin YL. Digestion rate of dietary starch affects systemic circulation of amino acids in weaned pigs. Br J Nutr (2010) 103(10):1404. doi: 10.1017/S0007114509993321 20102672

[16] Zhou XH He LQ Wan D Yang HS Yao K Wu GY. Methionine restriction on lipid metabolism and its possible mechanisms. Amino Acids (2016) 48(7):1533–40. doi: 10.1007/s00726-016-2247-7 27156065

[17] Xiao J Khan MZ Ma Y Alugongo GM Ma J Chen T. The antioxidant properties of selenium and vitamin e; their role in periparturient dairy cattle health regulation. Antioxidants (2021) 10:1555. doi: 10.3390/antiox10101555 34679690 PMC8532922

[18] He F Wu C Li P Li N Zhang D Zhu Q. Functions and signaling pathways of amino acids in intestinal inflammation. BioMed Res Int (2018) 2018:13. doi: 10.1155/2018/9171905 PMC5846438 29682569

[19] Andou A Hisamatsu T Okamoto S Chinen H Kamada N Kobayashi T. Dietary histidine ameliorates murine colitis by inhibition of pro-inflammatory cytokine production from macrophages. Gastroenterology (2009) 136(2):564–74. doi: 10.1053/j.gastro.2008.09.062 19027739

[20] Catanesi M Brandolini L d’Angelo M Benedetti E Tupone MG Alfonsetti M. L-methionine protects against oxidative stress and mitochondrial dysfunction in an *In vitro* model of Parkinson ‘s disease. Antioxidants (2021) 10(9):1467. 34573099 10.3390/antiox10091467 PMC8469212

[21] Mavrommatis A Mitsiopoulou C Christodoulou C Kariampa P Simoni M Righi F. Effects of supplementing rumen-protected methionine and lysine on milk performance and oxidative status of dairy ewes. Antioxidants (2021) 10(5):654. doi: 10.3390/antiox10050654 33922484 PMC8147003

[22] Ratika K James Singh KR Dahiya SS. Methionine, lysine and choline in dairy cows: a review article. Int J Curr Microbiol App Sci (2018) 7(7):3921–34. doi: 10.20546/ijcmas.2018.707.456

[23] Cho I-J Kim D Kim E-O Jegal K-H Kim J-K Park S-M. Cystine and methionine deficiency promotes ferroptosis by inducing b-cell translocation gene 1. Antioxidants (2021) 10:1543. doi: 10.3390/antiox10101543 34679678 PMC8532826

[24] Kung L Jr. Rode LM. Amino acid metabolism in ruminants. Anim Feed Sci Technol (1996) 59:167–72. doi: 10.1016/0377-8401(95)00897-7

[25] Castillo C Pereira V Abuelo A Hernández J. Effect of supplementation with antioxidants on the quality of bovine milk and meat production. Sci World J (2013), 616098. doi: 10.1155/2013/616098 PMC3856139 24348176

[26] Martínez Y Li X Liu G Peng Bin P Yan W Dairon M. The role of methionine on metabolism, oxidative stress, and diseases. Amino Acids (2017) 49(12):2091–8. doi: 10.1007/s00726-017-2494-2 28929442

[27] Girard CL Matte JJ. Effects of intramuscular injections of vitamin B12 on lactation performance of dairy cows fed dietary supplements of folic acid and rumen-protected methionine. J dairy Sci (2005) 88(2):671–6. doi: 10.3168/jds.S0022-0302(05)72731-4 15653534

[28] Pinotti L Baldi A Dell'Orto V. Comparative mammalian choline metabolism with emphasis on the high-yielding dairy cow. Nutr Res Rev (2002) 15(2):315–32. doi: 10.1079/NRR200247 19087410

[29] Drackley JK. Biology of dairy cows during the transition period: The final frontier? J dairy Sci (1999) 82(11):2259–73. doi: 10.3168/jds.S0022-0302(99)75474-3 10575597

[30] Trevisi E Amadori M Cogrossi S Razzuoli E Bertoni G. Metabolic stress and inflammatory response in high-yielding, periparturient dairy cows. Res Vet Sci (2012) 93:695–704. doi: 10.1016/j.rvsc.2011.11.008 22197526

[31] Bertoni G Trevisi E. Use of the liver activity index and other metabolic variables in the assessment of metabolic health in dairy herds. Vet Clin North Am Food Anim Pract (2013) 29(2):413–31. doi: 10.1016/j.cvfa.2013.04.004 23809898

[32] Brosnan JT Brosnan ME. The sulfur-containing amino acids: An overview. J Nutr (2006) 136:1636S–40S. doi: 10.1093/jn/136.6.1636S 16702333

[33] Martinov MV Vitvitsky VM Banerjee R Ataullakhanov FI. The logic of the hepatic methionine metabolic cycle. Biochim Biophys Acta (2010) 1804:89–96. doi: 10.1016/j.bbapap.2009.10.004 19833238 PMC2787964

[34] Lu SC Mato JM. S-adenosylmethionine in liver health, injury, and cancer. Physiol Rev (2012) 92:1515–42. doi: 10.1152/physrev.00047.2011 PMC3698976 23073625

[35] Preynat A Lapierre H Thivierge MC Palin MF Matte JJ Desrochers A. Effects of supplements of folic acid, vitamin B12, and rumen protected methionine on whole body metabolism of methionine and glucose in lactating dairy cows. J dairy sci (2009) 92:677–89. doi: 10.3168/jds.2008-1525 19164680

[36] Preynat A Lapierre H Thivierge MC Palin MF Cardinault N Matte JJ. Effects of supplementary folic acid and vitamin b (12) on hepatic metabolism of dairy cows according to methionine supply. J Dairy Sci (2010) 93:2130–42. doi: 10.3168/jds.2009-2796 20412928

[37] Garcia M Mamedova LK Barton B Bradford BJ. Choline regulates the function of bovine immune cells and alters the mRNA abundance of enzymes and receptors involved in its metabolism in vitro. Front Immunol (2018) 9:2448. doi: 10.3389/fimmu.2018.02448 30410489 PMC6211314

[38] Barak AJ Beckenhauer HC Junnila M Tuma DJ. Dietary betaine promotes generation of hepatic s-adenosylmethionine and protects the liver from ethanol-induced fatty infiltration. Alcohol Clin Exp Res (1993) 17:552–5. doi: 10.1111/j.1530-0277.1993.tb00798.x 8333583

[39] Fujii T Watanabe Y Fujimoto K Kawashima K. Expression of acetylcholine in lymphocytes and modulation of an independent lymphocytic cholinergic activity by immunological stimulation. Biog Amines (2002) 17:373–86. doi: 10.1163/15693910260698320

[40] Bionaz M Chen S Khan MJ Loor JJ. Functional role of PPARs in ruminants: Potential targets for fine-tuning metabolism during growth and lactation. PPAR Res (2013) 2013:684159. doi: 10.1155/2013/684159 23737762 PMC3657398

[41] Lopreiato V Mezzetti M Cattaneo L Ferronato G Minuti A Trevisi E. Role of nutraceuticals during the transition period of dairy cows: A review. J Anim Sci Biotechnol (2020) 11(1):1–18. doi: 10.1186/s40104-020-00501-x 32864127 PMC7450574

[42] Osorio JS Ji P Drackley JK Luchini D Loor JJ. Smartamine m and MetaSmart supplementation during the peripartal period alter hepatic expression of gene networks in 1-carbon metabolism, inflammation, oxidative stress, and the growth hormone-insulin-like growth factor 1 axis pathways. J Dairy Sci (2014) 97:7451–64. doi: 10.3168/jds.2014-8680 25282416

[43] Tsiplakou E Mavrommatis A Kalogeropoulos T Chatzikonstantinou M Koutsouli P Sotirakoglou K. The effect of dietary supplementation with rumen-protected methionine alone or in combination with rumen-protected choline and betaine on sheep milk and antioxidant capacity. J Anim Physiol Anim Nutr (2017) 101(5):1004–13. doi: 10.1111/jpn.12537 27278119

[44] Zhou Z Trevisi E Luchini DN Loor JJ. Differences in liver functionality indexes in peripartal dairy cows fed rumen-protected methionine or choline are associated with performance, oxidative stress status, and plasma amino acid profiles. J Dairy Sci (2017) 100(8):6720–32. doi: 10.3168/jds.2016-12299 28551192

[45] Vogt W. Oxidation of methionine residues in proteins: Tools, targets, and reversal. Free Rad Biol Med (1995) 18:93–105. doi: 10.1016/0891-5849(94)00158-G 7896176

[46] Stadtman ER Moskovitz J Berlett BS Levine RL. Cyclic oxidation and reduction of protein methionine residues is an important antioxidant mechanism. Mol Cell Biochem (2002) 234–235:3–9. doi: 10.1023/A:1015916831583 12162447

[47] Luo S Levine RL. Methionine in proteins defends against oxidative stress. FASEB J (2009) 23(2):464–72. doi: 10.1096/fj.08-118414 PMC2630790 18845767

[48] Han L Batistel F Ma Y Alharthi ASM Parys C Loor JJ. Methionine supply alters mammary gland antioxidant gene networks *via* phosphorylation of nuclear factor erythroid 2-like 2 (NFE2L2) protein in dairy cows during the periparturient period. J Dairy Sci (2018) 101:8505–12. doi: 10.3168/jds.2017-14206 29908802

[49] Coleman DN Lopreiato V Alharthi A Loor JJ. Amino acids and the regulation of oxidative stress and immune function in dairy cattle. J Anim Sci (2020) 98:S175–93. doi: 10.1093/jas/skaa138 PMC7433927 32810243

[50] Abbasi IHR Abbasi F Wang L Abd El Hack ME Swelum AA Hao R. Folate promotes s-adenosyl methionine reactions and the microbial methylation cycle and boosts ruminants production and reproduction. Amb Express (2018) 8(1):1–10. doi: 10.1186/s13568-018-0592-5 29687201 PMC5913057

[51] Abbasi IHR Abbasi F Soomro RN Abd El-Hack ME Abdel-Latif MA Li W. Considering choline as methionine precursor, lipoproteins transporter, hepatic promoter and antioxidant agent in animals. AMB Expr (2017) 7:214. doi: 10.1186/s13568-017-0513-z PMC5702286 29178045

[52] Yin J Li T Yin Y. Methionine and antioxidant potential. J Antioxid Act (2016) 1:12–7. doi: 10.14302/issn.2471-2140.jaa-16-1378

[53] Li P Yin YL Li D Kim SW Wu G. Amino acids and immune function. Br J Nutr (2007) 98:237–52. 12. doi: 10.1017/S000711450769936X 17403271

[54] Atmaca G. Antioxidant effects of sulfur-containing amino acids. Yonsei Med J (2004) 45(5):776–88. doi: 10.3349/ymj.2004.45.5.776 15515186

[55] Colovic MB Vasic VM Djuric DM Krstic DZ. Sulphur-containing amino acids: protective role against free radicals and heavy metals. Curr med Chem (2018) 25(3):324–35. doi: 10.2174/0929867324666170609075434 28595554

[56] Ye ZW Zhang J Townsend DM Tew KD. Oxidative stress, redox regulation and diseases of cellular differentiation. Biochim Biophys Acta (2015) 1850:1607–21. doi: 10.1016/j.bbagen.2014.11.010 PMC4433447 25445706

[57] Singhal SS Singh SP Singhal P Horne D Singhal J Awasthi S. Antioxidant role of glutathione stransferases: 4-hydroxynonenal, a key molecule in stress-mediated signaling. Toxicol Appl Pharmacol (2015) 289:361–70. doi: 10.1016/j.taap.2015.10.006 PMC4852854 26476300

[58] Galal AM Walker LA Khan IA. Induction of GST and related events by dietary phytochemicals: sources, chemistry, and possible contribution to chemoprevention. Curr Top Med Chem (2015) 14:2802–21. doi: 10.2174/1568026615666141208110721 25487008

[59] Zhou Z Loor JJ Piccioli-Cappelli F Librandi F Lobley GE Trevisi E. Circulating amino acids in blood plasma during the peripartal period in dairy cows with different liver functionality index. J Dairy Sci (2016) 99:2257–67. doi: 10.3168/jds.2015-9805 26778311

[60] Sun F Cao Y Cai C Li S Yu C Yao J. Regulation of nutritional metabolism in transition dairy cows: Energy homeostasis and health in response to post-ruminal choline and methionine. PloS One (2016) 11(8):e0160659. doi: 10.1371/journal.pone.0160659 27501393 PMC4976856

[61] Bartosz G. Non-enzymatic antioxidant capacity assays: limitations of use in biomedicine. Free Radical Res (2010) 44:711–20. doi: 10.3109/10715761003758114 20446897

[62] Mu T Kong GH Han ZY Li HX. Cytoprotection of methionine on hyperthermia-induced damage in bovine mammary epithelial cells. Cell Biol Int (2014) 38:971–6. doi: 10.1002/cbin.10271 24604888

[63] Han ZY Mu T Yang Z. Methionine protects against hyperthermia-induced cell injury in cultured bovine mammary epithelial cells. Cell Stress Chaperones (2015) 20:109–20. doi: 10.1007/s12192-014-0530-7 PMC4255250 25108357

[64] Zhou Z Bulgari O Vailati-Riboni M Trevisi E Ballou MA Cardoso FC. Rumen-protected methionine compared with rumen-protected choline improves immunometabolic status in dairy cows during the peripartal period. J dairy Sci (2016) 99(11):pp.8956–8969. doi: 10.3168/jds.2016-10986 27592438

[65] Ceciliani F Ceron JJ Eckersall PD Sauerwein H. Acute phase proteins in ruminants. J Proteomics (2012) 75:4207–31. doi: 10.1016/j.jprot.2012.04.004 22521269

[66] van der Vusse GJ. Albumin as fatty acid transporter. Drug Metab Pharmacokinet (2009) 24:300–7. doi: 10.2133/dmpk.24.300 19745557

[67] Osorio JS Trevisi E Ji P Drackley JK Luchini D Bertoni G. Biomarkers of inflammation, metabolism, and oxidative stress in blood, liver, and milk reveal a better immunometabolic status in peripartal cows supplemented with smartamine m or MetaSmart. J Dairy Sci (2014) 97:7437–50. doi: 10.3168/jds.2013-7679 25282419

[68] Batistel F Arroyo JM Garces CIM Trevisi E Parys C Ballou MA. Ethyl-cellulose rumen-protected methionine alleviates inflammation and oxidative stress and improves neutrophil function during the periparturient period and early lactation in Holstein dairy cows. J Dairy Sci (2018) 101:480–90. doi: 10.3168/jds.2017-13185 29103714

[69] Wang H Elsaadawy SA Wu Z Bu DP. Maternal supply of ruminally-protected lysine and methionine during close-up period enhances immunity and growth rate of neonatal calves. Front Vet Sci (2021) 8:780731. doi: 10.3389/fvets.2021.780731 34926646 PMC8677362

[70] Hu L Chen Y Cortes IM Coleman DN Dai H Liang Y. Supply of methionine and arginine alters phosphorylation of mechanistic target of rapamycin (mTOR), circadian clock proteins, and α-s1-casein abundance in bovine mammary epithelial cells. Food Funct (2020) 11(1):883–94. doi: 10.1039/C9FO02379H 31942894

[71] Dai H Coleman DN Hu L Martinez-Cortés I Wang M Parys C. Methionine and arginine supplementation alter inflammatory and oxidative stress responses during lipopolysaccharide challenge in bovine mammary epithelial cells *in vitro* . J dairy Sci (2020) 103(1):676–89. doi: 10.3168/jds.2019-16631 31733877

[72] Lee C Lobos NE Weiss WP. Effects of supplementing rumen-protected lysine and methionine during prepartum and postpartum periods on performance of dairy cows. J dairy Sci (2019) 102(12):11026–39. doi: 10.3168/jds.2019-17125 31548066

[73] Salama AA Caja G Albanell E Such X Casals R Plaixats J. Effects of dietary supplements of zinc-methionine on milk production, udder health and zinc metabolism in dairy goats. J Dairy Res (2003) 70(1):9–17. doi: 10.1017/S0022029902005708 12617388

[74] Abdelmegeid MK Vailati-Riboni M Alharthi A Batistel F Loor JJ. Supplemental methionine, choline, or taurine alter *in vitro* gene network expression of polymorphonuclear leukocytes from neonatal Holstein calves. J Dairy Sci (2017) 100:3155–65. doi: 10.3168/jds.2016-12025 28161165

[75] Zhou Z Ferdous F Montagner P Luchini DN Corrêa MN Loor JJ. Methionine and choline supply during the peripartal period alter polymorphonuclear leukocyte immune response and immunometabolic gene expression in Holstein cows. J Dairy Sci (2018) 101:10374–82. doi: 10.3168/jds.2018-14972 30172410

[76] Calamari L Soriani N Panella G Petrera F Minuti A Trevisi E. Rumination time around calving: An early signal to detect cows at greater risk of disease. J Dairy Sci (2014) 97:3635–47. doi: 10.3168/jds.2013-7709 24731630

[77] Zhou Z Vailati-Riboni M Luchini D Loor J. Methionine and choline supply during the periparturient period alter plasma amino acid and one-carbon metabolism profiles to various extents: Potential role in hepatic metabolism and antioxidant status. Nutrients (2016) 9:10. doi: 10.3390/nu9010010 28036059 PMC5295054

[78] Ospina PA McArt JA Overton TR Stokol T Nydam DV. Using nonesterified fatty acids and β-hydroxybutyrate concentrations during the transition period for herd-level monitoring of increased risk of disease and decreased reproductive and milking performance. Vet Clinics: Food Anim Pract (2013) 29(2):387–412. doi: 10.1016/j.cvfa.2013.04.003 23809897

[79] Zom RLG Van Baal J Goselink RMA Bakker JA De Veth MJ Van Vuuren AM. Effect of rumen-protected choline on performance, blood metabolites, and hepatic triacylglycerols of periparturient dairy cattle. J dairy Sci (2011) 94(8):4016–27. doi: 10.3168/jds.2011-4233 21787937

[80] Morrison EI Reinhardt H Leclerc H DeVries TJ LeBlanc SJ. Effect of rumen-protected b vitamins and choline supplementation on health, production, and reproduction in transition dairy cows. J dairy Sci (2018) 101(10):9016–27. doi: 10.3168/jds.2018-14663 30100511

[81] Bollatti JM Zenobi MG Barton BA Staples CR Santos JEP. Responses to rumen-protected choline in transition cows do not depend on prepartum body condition. J dairy Sci (2020) 103(3):2272–86. doi: 10.3168/jds.2019-17302 31882221

[82] Chung YH Brown NE Martinez CM Cassidy TW Varga GA. Effects of rumen-protected choline and dry propylene glycol on feed intake and blood parameters for Holstein dairy cows in early lactation. J Dairy Sci (2009) 92(6):2729–36. doi: 10.3168/jds.2008-1299 19448007

[83] Ster C Loiselle MC Lacasse P. Effect of postcalving serum nonesterified fatty acids concentration on the functionality of bovine immune cells. J Dairy Sci (2012) 95:708–17. doi: 10.3168/jds.2011-4695 22281335

[84] Pinotti L Polidori C Campagnoli A Dell’Orto V Baldi A. A meta-analysis of the effects of rumen protected choline supplementation on milk production in dairy cows. EEAP Sci Ser (2010) 127:321–2.

[85] Pinotti L Baldi A Politis I Rebucci R Sangalli L Dell’Orto V. Rumen protected choline administration to transition cows: effects on milk production and vitamin e status. J Vet Med Ser A (2003) 50:18–21. doi: 10.1046/j.1439-0442.2003.00502.x 12650504

[86] Cooke RF Silva Del Río N Caraviello DZ Bertics SJ Ramos MH Grummer RR. Supplemental choline for prevention and alleviation of fatty liver in dairy cattle. J Dairy Sci (2007) 90:2413–8. doi: 10.3168/jds.2006-028 17430945

[87] Elek P Gaál T Husvéth F. Influence of rumen-protected choline on liver composition and blood variables indicating energy balance in periparturient dairy cows. Acta Vet Hungarica (2013) 61(1):59–70. doi: 10.1556/avet.2012.053 23439292

[88] Shahsavari A Michael JD Al Jassim R. The role of rumen-protected choline in hepatic function and performance of transition dairy cows. Br J Nutr (2016) 116(1):35–44. doi: 10.1017/S0007114516001641 27138530

[89] Goselink RMA van Baal J Widjaja HCA Dekker RA Zom RLG de Veth MJ. Effect of rumen-protected choline supplementation on liver and adipose gene expression during the transition period in dairy cattle. J Dairy Sci (2013) 96(2):1102–16. doi: 10.3168/jds.2012-5396 23200476

[90] Baumgard LH Rhoads RP Jr. Effects of heat stress on postabsorptive metabolism and energetics. Annu Rev Anim Biosci (2013) 1(1):311–37. doi: 10.1146/annurev-animal-031412-103644 25387022

[91] Ouellet V Cabrera VE Fadul-Pacheco L Charbonneau É. The relationship between the number of consecutive days with heat stress and milk production of Holstein dairy cows raised in a humid continental climate. J dairy Sci (2019) 102(9):8537–45. doi: 10.3168/jds.2018-16060 31255266

[92] Holdorf HT White HM. Effects of rumen-protected choline supplementation in Holstein dairy cows during electric heat blanket-induced heat stress. J Dairy Sci (2021) 104(9):9715–25. doi: 10.3168/jds.2020-19794 34127269

[93] Zenobi MG Gardinal R Zuniga JE Mamedova LK Driver JP Barton BA. Effect of prepartum energy intake and supplementation with ruminally protected choline on innate and adaptive immunity of multiparous Holstein cows. J dairy Sci (2020) 103(3):2200–16. doi: 10.3168/jds.2019-17378 31954584

[94] Zenobi MG Scheffler TL Zuniga JE Poindexter MB Campagna SR Gonzalez HC. Feeding increasing amounts of ruminally protected choline decreased fatty liver in nonlactating, pregnant Holstein cows in negative energy status. J dairy Sci (2018) 101(7):5902–23. doi: 10.3168/jds.2017-13973 29680650

[95] McFadden JW Girard CL Tao S Zhou Z Bernard JK Duplessis M. Symposium review: One-carbon metabolism and methyl donor nutrition in the dairy cow. J dairy Sci (2020) 103(6):5668–83. doi: 10.3168/jds.2019-17319 32278559

[96] da Silva RP Kelly KB Lewis ED Leonard KA Goruk S Curtis JM. Choline deficiency impairs intestinal lipid metabolism in the lactating rat. J Nutr Biochem (2015) 26(10):1077–83. doi: 10.1016/j.jnutbio.2015.04.015 26092371

[97] Jacometo CB Alharthi AS Zhou Z Luchini D Loor JJ. Maternal supply of methionine during late pregnancy is associated with changes in immune function and abundance of microRNA and mRNA in Holstein calf polymorphonuclear leukocytes. J dairy Sci (2018) 101(9):8146–58. doi: 10.3168/jds.2018-14428 29908814

[98] Tsiplakou E Mavrommatis A Skliros D Righi F Flemetakis E. The impact of rumen-protected amino acids on the expression of key-genes involved in the innate immunity of dairy sheep. PloS One (2020) 15(5):e0233192. doi: 10.1371/journal.pone.0233192 32407360 PMC7224535

[99] Li Y Bi Y Diao Q Piao M Wang B Kong F. The limiting sequence and appropriate amino acid ratio of lysine, methionine, and threonine for seven-to nine-Month-Old Holstein heifers fed corn–soybean m-based diet. Animals (2019) 9(10):750. doi: 10.3390/ani9100750 31574931 PMC6827085

[100] Liang Y Batistel F Parys C Loor JJ. Glutathione metabolism and nuclear factor erythroid 2-like 2 (NFE2L2)- related proteins in adipose tissue are altered by supply of ethyl-cellulose rumen-protected methionine in peripartal Holstein cows. J Dairy Sci (2019) 102:5530–41. doi: 10.3168/jds.2018-15687 30954259

[101] Fagundes MA Yang SY Eun JS Hall JO Moon JO Park JS. Influence of supplementing a methionine derivative, n-acetyl-l-methionine, in dairy diets on production and ruminal fermentation by lactating cows during early to mid lactation. J dairy Sci (2018) 101(8):7082–94. doi: 10.3168/jds.2017-14130 29729912

[102] Lopreiato V Vailati-Riboni M Bellingeri A Khan I Farina G Parys C. Inflammation and oxidative stress transcription profiles due to *in vitro* supply of methionine with or without choline in unstimulated blood polymorphonuclear leukocytes from lactating Holstein cows. J Dairy Sci (2019) 102(1):10395–410. doi: 10.3168/jds.2019-16413 31447151

[103] Osorio JS Ji P Drackley JK Luchini D Loor JJ. Supplemental smartamine m or MetaSmart during the transition period benefits postpartal cow performance and blood neutrophil function. J Dairy Sci (2013) 96:6248–63. doi: 10.3168/jds.2012-5790 23910549

[104] Vailati-Riboni M Zhou Z Jacometo CB Minuti A Trevisi E Luchini DN. Supplementation with rumen-protected methionine or choline during the transition period influences whole-blood immune response in periparturient dairy cows. J Dairy Sci (2017) 100:3958–68. doi: 10.3168/jds.2016-11812 28318590

[105] Zhou Z Garrow TA Dong X Luchini DN Loor JJ. Hepatic activity and transcription of betaine-homocysteine methyltransferase, methionine synthase, and cystathionine synthase in periparturient dairy cows are altered to different extents by supply of methionine and choline. J Nutr (2017) 147:11–9. doi: 10.3945/jn.116.240234 27881594

[106] Alharthi AS Coleman DN Liang Y Batistel F Elolimy AA Yambao RC. Hepatic 1-carbon metabolism enzyme activity, intermediate metabolites, and growth in neonatal Holstein dairy calves are altered by maternal supply of methionine during late pregnancy. J Dairy Sci (2019) 102:10291–303. doi: 10.3168/jds.2019-16562 31477291

[107] Guretzky NJ Carlson DB Garrett JE Drackley JK. Lipid metabolite profiles and milk production for Holstein and Jersey cows fed rumen-protected choline during the periparturient period. J dairy Sci (2006) 89(1):188–200. doi: 10.3168/jds.S0022-0302(06)72083-5 16357282

[108] Bollatti JM Zenobi MG Artusso NA Alfaro GF Lopez AM Barton BA. Timing of initiation and duration of feeding rumen-protected choline affects performance of lactating Holstein cows. J dairy Sci (2020) 103(5):4174–91. doi: 10.3168/jds.2019-17293 32171515

[109] Salama AA Duque M Wang L Shahzad K Olivera M Loor JJ. Enhanced supply of methionine or arginine alters mechanistic target of rapamycin signaling proteins, messenger RNA, and microRNA abundance in heat-stressed bovine mammary epithelial cells in vitro. Journal of dairy science (2019) 102(3):2469–80. doi: 10.3168/jds.2018-15219 30639019

[110] Wang JX Zhang Q Pei S Yang B. Effect and mechanism of miR-34a on proliferation, apoptosis and invasion of laryngeal carcinoma cells. Asian Pac J Trop Med (2016) 9:494–8. doi: 10.1016/j.apjtm.2016.03.018 27261861

[111] Jena MK. MicroRNAs in the development and neoplasia of the mammary gland. F1000Res (2017) 6:1018. doi: 10.12688/f1000research.12005.2. 28979765 PMC5609084

[112] Ma YF Batistel F Xu TL Han LQ Bucktrout R Liang Y. Phosphorylation of AKT serine/threonine kinase and abundance of milk protein synthesis gene networks in mammary tissue in response to supply of methionine in periparturient Holstein cows. J dairy Sci (2019) 102(5):4264–74. doi: 10.3168/jds.2018-15451 30879806

[113] Nan X Bu D Li X Wang J Wei H Hu H. Ratio of lysine to methionine alters expression of genes involved in milk protein transcription and translation and mTOR phosphorylation in bovine mammary cells. Physiol Genomics (2014) 46:268–75. doi: 10.1152/physiolgenomics.00119.2013 24474444

[114] Dong X Zhou Z Saremi B Helmbrecht A Wang Z Loor JJ. Varying the ratio of lys: Met while maintaining the ratios of thr: Phe, lys: Thr, lys: His, and lys: Val alters mammary cellular metabolites, mammalian target of rapamycin signaling, and gene transcription. J Dairy Sci (2018) 101:1708–18. doi: 10.3168/jds.2017-13351 29248224

[115] Jacometo CB Zhou Z Luchini D Corrêa MN Loor JJ. Maternal supplementation with rumen-protected methionine increases prepartal plasma methionine concentration and alters hepatic mRNA abundance of 1-carbon, methionine, and transsulfuration pathways in neonatal Holstein calves. J Dairy Sci (2017) 100(4):3209–19. doi: 10.3168/jds.2016-11656 28161170

[116] Neumann S Razen M Habermehl P Meyer CU Zepp F Kirkpatrick CJ. The non-neuronal cholinergic system in peripheral blood cells: effects of nicotinic and muscarinic receptor antagonists on phagocytosis, respiratory burst and migration. Life Sci (2007) 80:2361–4. doi: 10.1016/j.lfs.2007.01.010 17286990

[117] Tsiplakou E Mavrommatis A Skliros D Sotirakoglou K Flemetakis E Zervas G. The effects of dietary supplementation with rumen-protected amino acids on the expression of several genes involved in the immune system of dairy sheep. J Anim Physiol Anim Nutr (Berl) (2018) 102(6):1437–49. doi: 10.1111/jpn.12968 30043476

[118] Osorio JS Jacometo CB Zhou Z Luchini D Cardoso FC Loor JJ. Hepatic global DNA and peroxisome proliferator-activated receptor alpha promoter methylation are altered in peripartal dairy cows fed rumen-protected methionine. J dairy Sci (2016) 99(1):234–44. doi: 10.3168/jds.2015-10157 26585478

[119] Palombo V Alharthi A Batistel F Parys C Guyader J Trevisi E. Unique adaptations in neonatal hepatic transcriptome, nutrient signaling, and one-carbon metabolism in response to feeding ethyl cellulose rumen-protected methionine during late-gestation in Holstein cows. BMC Genomics (2021) 22(1):1–24. doi: 10.1186/s12864-021-07538-w 33865335 PMC8053294

